# Not just bricks and mortar: planning hospital cancer services for Aboriginal people

**DOI:** 10.1186/1756-0500-4-62

**Published:** 2011-03-14

**Authors:** Sandra C Thompson, Shaouli Shahid, Dawn Bessarab, Angela Durey, Patricia M Davidson

**Affiliations:** 1 Winthrop Professor, Chair in Rural Health and Director, Combined Universities Centre for Rural Health, University of Western Australia, 167 Fitzgerald St, Geraldton, Western Australia 6530 Australia; 2 Adjunct Professor, Centre for International Health, Curtin University, GPO Box U1987 Perth 6845 Australia; 3 Post-doctoral Research Fellow, The Western Australian Centre for Cancer and Palliative Care and the Aboriginal Health Education and Research Group, Curtin Health Innovation Research Institute, Curtin University, Shenton Park Campus 10 Selby St Shenton Park 6008 WA Australia; 4 Associate Professor, Aboriginal Health Education and Research Group, Curtin Health Innovation Research Institute, Curtin University, Kent Street, Bentley, Perth, Western Australia. 6102. Australia; 5 Senior Research Fellow, Aboriginal Health Education and Research Group, Curtin Health Innovation Research Institute, Curtin University, Kent Street, Bentley, Western Australia. 6102. Australia; 6 Centre for Cardiovascular and Chronic Care, University of Technology Sydney and Curtin University, Jones St, Broadway, 2007 NSW. Australia

## Abstract

**Background:**

Aboriginal people in Australia experience higher mortality from cancer compared with non-Aboriginal Australians, despite an overall lower incidence. A notable contributor to this disparity is that many Aboriginal people do not take up or continue with cancer treatment which almost always occurs within major hospitals.

Thirty in-depth interviews with urban, rural and remote Aboriginal people affected by cancer were conducted between March 2006 and September 2007. Interviews explored participants' beliefs about cancer and experiences of cancer care and were audio-recorded, transcribed verbatim and coded independently by two researchers. NVivo7 software was used to assist data management and analysis. Information from interviews relevant to hospital services including and building design was extracted.

**Findings:**

Relationships and respect emerged as crucial considerations of participants although many aspects of the hospital environment were seen as influencing the delivery of care. Five themes describing concerns about the hospital environment emerged: (i) being alone and lost in a big, alien and inflexible system; (ii) failure of open communication, delays and inefficiency in the system; (iii) practicalities: costs, transportation, community and family responsibilities; (iv) the need for Aboriginal support persons; and (v) connection to the community.

**Conclusions:**

Design considerations and were identified but more important than the building itself was the critical need to build trust in health services. Promotion of cultural safety, support for Aboriginal family structures and respecting the importance of place and community to Aboriginal patients are crucial in improving cancer outcomes.

## Background

Aboriginal people in Australia have poorer health outcomes across the lifespan and a wide range of conditions. The reasons for this are complex and multifaceted and include a complex interplay of biological, social and psychological factors. However, for cancer outcomes, it is not because Aboriginal people have a higher incidence of cancer *per se*, but for other reasons that they experience inferior outcomes compared to non-Aboriginal Australians. They have lower participation in cancer screening programs, cancer is commonly diagnosed at a later stage and there are higher rates of cancers which have a poorer prognosis, such as lung, liver and cervical cancer [1-3]. There is evidence that rural and remote Aboriginal people have poorer outcomes [3-6] and the failure in health service delivery is evidenced by the poorer outcomes for Aboriginal compared to non-Aboriginal people for almost any cancer diagnosed with equivalent staging. Thus, despite Australia having relevant services, specialists and a universal health insurance system, challenges remain in facilitating Aboriginal entry into and ongoing participation in treatment for those affected by cancer. This situation should cause those involved in health service planning and service delivery to consider how cancer health services could better address the cancer treatment needs of Aboriginal people. Service improvements are needed to encourage uptake [7].

Recent research findings suggest that Aboriginal Australians with cancer have multiple reasons for their reluctance to attend cancer services. These include discrimination and lack of respect and understanding of Aboriginal culture shown by health care providers [8], a preference for traditional healing methods over western medicine [9], beliefs that cancer is a death sentence, contagious, shameful, or related to payback and the dislocation from 'country' or homeland to access treatment. Many Aboriginal patients find hospitals alienating and frightening with inflexible and discriminatory methods of care [8]. To improve Aboriginal cancer outcomes, there is an imperative to meet the needs of Aboriginal people including the 'physical, psychological, social, cultural and spiritual' aspects of health care [10-13] that are integral to the healing process. These needs to be reflected in service delivery and design of the service environment.

In 2005, the Western Australian Cancer and Palliative Care Network produced the Western Australian Cancer Services Framework. This stated that that "the agreed standard is that highly specialised cancer services are provided at a limited number of sites to ensure that expertise is maintained and the outcomes are optimal. Cancer Centres are proposed at tertiary sites with Cancer Units at some hospital facilities and Outreach Programs to other regional and rural locations"[14]. More recently the Australian Government has announced significant investment in cancer units in rural areas to support chemotherapy treatment closer to home for rural residents.

This paper responds to question posed by planners committed to establishing a Cancer Unit to meet the needs of Aboriginal people. The initial analysis occurred when an outer urban hospital was being upgraded to incorporate a Cancer Unit and health planners wanted to ensure that health care delivery would better meet the needs of Aboriginal people, provide a welcoming environment and increase Aboriginal people's use of, and satisfaction with services. Given that the hospital is located in an urban growth corridor, the planners expected it to become a tertiary hospital in approximately 10 years. As researchers undertaking qualitative research exploring Aboriginal people's beliefs and understandings of cancer and their experiences of cancer care, we were invited to be part of a consultation around planning the building of such a cancer service and draw on our research findings to inform this process.

This paper utilises interviews with Aboriginal patients that aimed to identify what helped or impeded Aboriginal participation in cancer care. The paper will link these findings to argue the importance not only of care that is respectful of Aboriginal needs, but also of a hospital cancer unit design features that Aboriginal patients consider conducive to the healing process. Our purpose is to provide evidence from research to assist those planning cancer treatment services to ensure they meet the needs of Aboriginal people.

## Methods

### Consultation process

The research informing this design project involved a systematic collection of data, including interviews, from a range of individuals where findings were presented in a considered and rigorous manner[9,15]. Detailed information included efforts to build trust and engage respectfully with Aboriginal community members. Such rich information was unlikely to be gleaned from a two-hour community meeting at which the voices of Aboriginal people may not be adequately heard, even *if *they did attend and actively participated. However, one-off meetings of this type may not be held at a time or place suited to Aboriginal stakeholders so their insights into cancer services by reason of their own or close family members' personal experience of cancer, may not be forthcoming. What is offered as a genuine opportunity for Aboriginal engagement by health planners might be considered as tokenistic by those concerned about meaningful and respectful Aboriginal consultation.

In responding to the request to provide information, we first advised the planners of the need to have direct input from Aboriginal people. Consulting with key stakeholders in the Aboriginal community is a crucial part of the planning process to identify cultural needs and ensure they are met within the parameters of the service delivery and building project [7]. A daytime meeting was convened, and attended by a number of Aboriginal people (females only) who were all involved in health care delivery, including two who were Indigenous program officers within the population screening programs for cervical and breast cancer. In addition, the planners invited Aboriginal people (along with the general population) to have input through a Community Forum held one evening at a Community Centre where senior officials of the Department of Health involved in the planning process were to attend to listen to community views. Community members were also invited to send comments to the planning group via e-mail. The question posed by the planners was "From the perspective of Aboriginal cancer patients, what are the issues that you believe need to be addressed?" They were also interested in knowing about how the Aboriginal community wanted to be consulted on these issues as the project unfolded, a question that could only be responded to by the Aboriginal community.

The study was approved by four Human Research Ethics Committees, including the Western Australian Aboriginal Health Information and Ethics Committee.

### Data collection

The aim of the broad study of which this was part was to identify Aboriginal people's views of the factors affecting Aboriginal participation in cancer care including their beliefs about cancer and experiences of cancer care. Briefly, 30 in-depth interviews with urban, rural and remote Aboriginal people affected by cancer were conducted between March 2006 and September 2007. Recruitment occurred through key contacts of the researchers, community organisations working in Aboriginal health and via health professionals in primary or tertiary care, with snowball recruitment where initial participants recommended others as candidates for the study. Interviewees were adults (7 males, 23 females) from urban (11) and rural (19) areas and included Aboriginal cancer patients and survivors (14) and family members (16) of people with cancer or who had died from cancer. All participants could speak English and gave written informed consent. Interviews were audio-recorded, transcribed verbatim and coded independently by two researchers.

### Data analysis

NVivo7 software was used to assist data management and analysis. Participants' narratives were divided into broad categories to allow identification of key themes and discussed by the research team. Feedback sessions with available participants assisted clarification of whether emerging themes were an accurate reflection of participants' experiences.

For the purpose of this paper, information from interviews relevant to buildings and other aspects of service planning was extracted, discussed within the research team and presented at a meeting with service planners which included the participation of a number of Aboriginal people. The major themes are presented below and then discussed in the light of existing literature around hospital design features around culturally competent care and only with this report focusing mainly upon how these findings should be interpreted in cancer service planning.

### Recurring Themes

It is important firstly to acknowledge the diversity of Aboriginal peoples and therefore that a range of views will be expressed on any topic. Our analysis concentrates on recurring themes expressed by many or a group of participants.

Emerging from the data were five discrete but interrelated themes describing concerns about the hospital environment as expressed by Aboriginal people: (i) being alone and lost in a big, alien and inflexible system; (ii) failure of openness, delays and inefficiency; (iii) practicalities: costs, transportation, community and family responsibilities, (iv) the need for Aboriginal support persons; and (v) connection back to their community. Thus, instrumental drivers in participants' decisions to access and negotiate with the health care system were family and support systems and although the hospital environment was important, it was relationships that emerged as crucial to access.

#### Alone and Lost: A Big, Alien and Inflexible System

Aboriginal participants reported finding the medical system cold, indifferent and as unwilling to tolerate approaches to treatment that were considered different to the traditional biomedical model. Many reported the absence of warm interpersonal interaction which left them feeling that staff were insensitive and lacking in warmth. Comments made in relation to cancer services were the need for big hospitals to have more understanding and compassion about people's situation. The current services were considered too inflexible and formal to function well for Aboriginal people. For example, being "moved around" by the medical system contributed to the environment being considered cold and isolating. This accentuated the disconnection from 'place' and cultural places that was associated with the hospital and therefore isolation from country and home.

The alienating environment reported by most participants was felt most acutely by those from rural and remote areas and contrasted with their sense of belonging in the bush and close bond to country. The sheer size of cancer treatment services in tertiary hospital settings was unfamiliar and overwhelming to people from rural and remote areas and created difficulties:

*The first time they have probably even been to Perth [capital city of Western Australia] is to go down to a big square hospital, a big cement building. Is it any wonder they die? *(Rural female family member)

Another participant described the hospital as one big grey building stating that "*We were just about crying and so was he [my dad]. He wanted to get back to the bush." *Being given a map of the hospital was not helpful; to rural participants, the tertiary hospital itself was a big city.

Low literacy was acknowledged as another challenge observed by participants, even when it didn't directly impact upon them. For example, one participant described that although she felt she was okay because she could read, it was difficult for others without these skills. She considered that hospital staff were often unaware of these challenges:

*... you know some of the elders and some young people who are stressed out and walking around trying to find B block or whatever, the radiation centre, yeah; it's hard. If the line is not there or the name is not there, you can't find the place. This is the hospital*. (Urban female family member)

The hospital was thus seen as symbolising white dominance, where Aboriginal cultural needs were neither recognized nor met. The absence of a welcoming environment was evident from the time participants first arrived, lacking symbols that showed respect of Aboriginal culture such as appropriate photos, flags, and signage.

Many criticisms of current services were that they did not cater for visits from a big family nor did staff understand the importance of this, a theme reiterated by most participants. The importance of family support and having children and grandchildren around at a time of great stress was in conflict with hospital policies such as only allowing two visitors at a time and being generally unwelcoming of big families and was not reflected in the design of the building. For patients, this often meant they did not feel comfortable or secure there and that hospitals were not adequately supportive of their needs:

*Imagine if that was, you know, an Aboriginal women from one of the remote communities who couldn't have her family down by her side, you know, to be with her and to talk with doctors and, you know, to explain what was happening*. (Urban, female family member)

The problems encountered in hospitals were in contrast to that described by one participant who was appreciative of the arrangements of a particular hospice and how it embraced family involvement:

*They supported us as a family in every way. For if we wanted to stay over, we could stay over. They have got a kitchen...every family member of the patient ...they could use that any time day and night, 24 hour services. There was no restriction. All you have to do is go and ring the bell at 2 o'clock and you are in...and my family in the last two weeks of her life basically lived there and had rosters off. She was never left alone*. (Urban, female family member)

This suggests that a design that enables family members to be self catering and cook for themselves enables a familiarity and homeliness that helped with them feeling more comfortable and welcome.

The importance of family involvement was frequently reiterated:

"*Yeah, well, that's our culture. Like, when someone is close to passing away the whole extended family will come. That's been like that for years and years. You can't change that, you know" *(Urban, female patient).

At a time of great stress and uncertainty about their future, Aboriginal people with a cancer diagnosis expressed that they wanted their family close. Enabling large Aboriginal families to be involved in providing care and support to their family member was a key issue raised in relation to hospital-based care. While hospital design and policy could help with this, broader inter-sectoral collaboration is needed to ensure health services respectful of Aboriginal cultural needs [7].

Participants also commented on frequent staff turnover. Personal relationships are important for Aboriginal people, and multiple changes of staff and lack of continuity of care was considered to indicate a lack of caring. Other comments were that there was too much writing, that the care was too systematic rather than personal "*you are treated as numbers*", and that the approach was theory-based rather than person-centred.

While not universally discussed, a number of participants mentioned the desire for traditional Aboriginal medicine and healers as playing an important role alongside more "western" medical interventions. The separation from country and those with similar cultural beliefs contributed to this, but opportunities to use ceremonies such a smoking to cleanse the spirit and access to gardens in which plants considered to have medicinal or other significance could be considered. Not liking the food was often raised, and again both design and service arrangements could be adopted that helped address this issue.

#### Failure to be Open, Delays and Inefficiency

It is important to appreciate the context of distrust and dislike many Aboriginal people have of western institutions [16]. Many participants reported their frustration with delays in the hospital system. Given the aversion that most people expressed to being in hospital, their distrust of them and desire to return home to family and country, delays in treatment and discharge were considered particularly significant. Participants commented that they felt their own and their family members' concerns were not listened to or acknowledged by staff, with some reporting an unwillingness of medical staff to be forthcoming about the severity and prognosis of their illness:

*They would upset her and she would ring us and say, 'Can you come in? The doctor has just been in'. Then we would ask her, 'Well, what's wrong? You know, what did the doctor say?' 'Oh, I don't know; I can't remember', yeah, because they talk in technical terms, you know, medical terms. Patients can't understand medical terms*. (Urban, female family member)

Communication emerged as a major issue in the context of cancer care in the hospital setting and has been reported in detail elsewhere [11]. While for a proportion of Aboriginal people English is not their first language, the above extract highlights that jargon is yet another barrier to communication. Given the dislike that many Aboriginal people expressed of being in an alien hospital environment and their desire to return to family and country, communication issues are of heightened importance. For example, what may be considered within modern hospital practice as risk management and good care in terms of checking and re-checking was reported by some participants as being "*inefficient staff*" and "*management problems*". This epitomises a risk management approach in which the priorities of management are dominant, with failure to consult or act upon the priorities of Aboriginal people. The experience of hospital based care in such instances, is unlikely to be addressed by hospital "design" in a conventional sense, and requires a different approach to improving the Aboriginal person's experience of cancer treatment. This approach needs to be at multiple levels to improve many aspects of an Aboriginal person's experience of cancer treatment and include design measures that ensure the hospital environment is more conducive to healing.

#### Practicalities: Cost, Transportation, Community and Family Responsibilities

Managing the practical issues of daily life such as money, transportation, community responsibilities and child care needs affected people's ability to attend clinics and follow up on treatment. Financial issues and the expense of treatment were other concerns raised by all participants, as was cultural safety. The cost and availability of safe and Aboriginal-friendly accommodation was also important for rural patients. Negotiating getting to and within the hospital system, understanding what is happening and likely treatment options were regarded as vital to addressing the key barriers to Aboriginal people entering the hospital system:

*...especially a lot of people when they come from the community, they don't know what they are gonna face. Yeah, like... where they are gonna stay, how they are gonna support themselves for six weeks, and if their family is gonna come. And...A lot of support should come from within the hospital as well, especially social workers, if people need it*. (Remote, male patient)

A consequence of not addressing these needs may be failure of Aboriginal people to take up or continue with treatment. As one female family member living in Perth said, "*I know of Aboriginal women like from remote communities who have made decisions not to come down to Perth for treatment because of the way that they are treated here." *This illustrates the way that stories about bad experiences of Aboriginal people are talked about and shared.

#### The Need for Aboriginal Support Persons

Another recurring theme raised by Aboriginal people was the need to have Aboriginal support people among the health care team - simply the inclusion of Aboriginal people in the health care team would equate the service as being more welcoming. Participants spoke of how alienating and disempowering the hospital environments was for Aboriginal people, with them often lacking the confidence to speak up. The health care team was seen as only treating the disease and not adapting the model of care to suit Aboriginal patients *"they want us to fit into their culture. They want to take us from our box and put us in their box" *(urban, female family member), yet participants craved understanding about their needs, in an environment that was caring and friendly. It was believed that Aboriginal staff would have sensitivity and understanding and that simply spending time with them would show empathy. Underlying this was a belief that relationship is important and Aboriginal patients want one-to-one contact with someone who genuinely cares about them. Aboriginal liaison officers were seen as facilitating logistical aspects of care and assisting Aboriginal patients with negotiating the hospital system, understanding what is happening and likely treatment options, and addressing the key barriers to Aboriginal people entering the hospital system, a theme that has been identified in other research [12,13,17,18]. Aboriginal support staff could also advise about resources that were available and help with the logistical needs of people coming to the city for treatment. The importance of an Aboriginal staff member within the health care team and the many different roles that they can fulfil includes ensuring that patients understand treatment plans, possible outcomes and responsibilities and that medical staff are culturally sensitised and can deal with patients and their families sensitively and with compassion.

#### Connection to the Community

Many respondents felt that hospital treatment should be more connected with the community as part of a broader Aboriginal outreach program incorporating communication, support, community contact and relationship-building activities and initiatives. A presence in the community was seen as an important component of educating people about available services. Involving the Aboriginal community would help spread the word and being seen actively doing something was important in gaining the trust of the Aboriginal people. Part of increasing people's willingness to use and access the hospital was likely to be increased by hearing stories about individuals' experiences in the hospitals, and that their treatment was good. A major message was to make time for people and to use information from local sources to ensure appropriate supportive care. Having staff who worked between the hospital and the community was proposed, with providing resources and facilities for more than just medical treatments considered as important to build trust and confidence with communities.

## Discussion

### Potential solutions and currently effective programmes

Although the impetus for this analysis arose from planning a new building to be more welcoming and respectful of Aboriginal culture, the explicit focus of our research was on people's experience of cancer care, and factors that supported or impeded their participation in such care. While some responses included suggestions on how to make the built environment more welcoming and friendly (Table 1), concerns related to hospital design and building were less prominent in participants responses than their perceptions of respect, understanding and cultural safety in service delivery. Nonetheless, these perceived needs need to be reflected in designing buildings and services if positive outcomes are to be achieved [7]. Our findings provide rich information that can inform hospital design and service delivery to be more culturally respectful in ways that can increase satisfaction and improve attendance rates. Aboriginal stakeholders are key players in the process. Weaving participants' primary concerns about dislocation from family and country and negotiating a complex and alienating environment are key considerations in the design and planning process of all aspects of care.

**Table 1:** Recommendations for Physical Design of Hospitals

Locate an Aboriginal Welcome Desk at the entrance to the hospital staffed by with Aboriginal people to allow a welcome/acknowledgement of country and to assist patients and their family members effectively negotiate the hospital system
Create a physical environment that provides a welcoming signal; consider colour, texture, light, form and cultural symbolism to create a more comforting and homelike environment

Improve signage: recognise that some people have limitations in literacy, eyesight and physical fitness

Consider a building aesthetic that is sympathetic to Aboriginal people in its ambience, enabling views over or connection to Australian bushland

Build hospital rooms of adequate size to allow visits from large families and spaces that facilitate the inclusion of family members in care planning

Provide options for affordable childcare within hospitals and near Aboriginal hostels consider privacy, confidentiality and gender sensitivities in planning

Ensure there is the opportunity for family members to stay in safe, low cost accommodation close to the treatment centre

Consider accessibility to the service by public transport and availability of minimal cost parking

Acknowledge that elevators and high technology machines will be outside the experience of some Aboriginal people and need to be explained

Adopt design features that support person-centred relationship-based care rather than remote monitoring

Ensure ready access to information that helps cross cultural understanding where it is needed (maps with the location of remote communities, language groups, availability and contacts for interpreters for service providers; suitable resources to explain cancer and cancer treatments to Aboriginal people).

While it is well documented that environmental factors can increase anxiety and stress [16], buildings and services can be designed to be less intimidating, more culturally respectful and conducive to healing thereby increasing patient satisfaction and attendance rates [7,19]. Many respondents described the hospital as a big and unfriendly environment - symbolised by the building itself - and a number of people felt alone and lost when they first arrived. In at least one tertiary hospital in another state, an Aboriginal Health Unit, "a place where you can come for a yarn, cuppa or just sit quietly" is centrally located and "committed to providing any support possible in assisting Aboriginal and Torres Strait Islanders and their families" [20]. This service can welcome Aboriginal people from elsewhere, as well as provide a presence and friendly face to assist orientation and direction to the relevant department of service. Its prominent location provides important symbolism that Aboriginal people are welcome and that Aboriginal people work at the hospital, as well as offering a practical service through the availability of advice from people who understand Aboriginal people and the fears that they might be experiencing on such a visit.

In terms of hospital care planning, Aboriginal families want to be able to support their loved ones when they are sick and away from their home - particularly important given the reputation that many mainstream hospitals have for not providing culturally safe care [21]. These sentiments echo those reported by others [22,23] and are not unique to Aboriginal patients [24,25]. It is institutional policies, organisational culture and personal interaction as much as space that make Aboriginal families feel unwelcome. However, incorporating cultural elements into the design process is also important as many hospital rooms may currently be too small to accommodate extended Aboriginal families. In the course of the research many participants commented that non-Aboriginal service providers may not understand the significance of restricting visits to the immediate family given that an Aboriginal person might have raised nieces and nephews who are considered as his or her kids too. Our findings suggest that restricted visiting add to the stress of family illness and can create friction in the family. Moreover, there is no service that assists with finding accommodation for visiting Aboriginal families, many of whom have travelled considerable distances by car over several days to provide the emotional and caring support for the person affected by cancer. Even if provision of accommodation for extended family is beyond the remit of a hospital Cancer Unit, it is important for Aboriginal patients' cancer care to plan for co-location of reasonable and affordable accommodation for families so they can help support the patient in their cancer treatment. In discussion with people who have been involved in planning new hospitals about these findings, concerns were expressed that even when the need for more room is recognised early on in the hospital planning process, a preoccupation with escalating hospital building costs may see room sizes reduced back to the bare functional minimum. Such changes neglect important aspects of psychosocial care in a way that impacts particularly hard upon Aboriginal patients. It highlights the tension between the economic bottom line with a 'functional minimum', and creating a hospital environment that promotes healing for Aboriginal patients -the irony being that in the longer term it may add to health care costs and accentuate disparities in health outcomes.

Recurring direct and oblique references were made by many participants about their love of the bush and its importance to them. Often this was related with the area from which they had come and the association of bush with home, even for those currently living in urban areas. However, given Aboriginal people's connection to and love of Australian landscape, it would seem reasonable to plan for services that allow patients visual and physical access to an inviting bush landscape. Wilcannia Hospital in New South Wales has been designed to suit local Aboriginal people, and has been lauded as effectively engaging the community in planning and building. The building has worked to effectively to incorporate the Darling River which has spiritual and cultural significance for the local Barkindji people both in its architecture and by views of the river patients have from their rooms [26]. Aboriginal-focussed design was undoubtedly not the only remit of those involved in this Hospital Cancer Unit planning, but possibilities for a design that embraces nature and is sympathetic with the natural environment should be considered, arguably for the benefit of all patients not just for Aboriginal people [19]. There is good evidence that access to views of the natural environment can help reduce hospital stays and the need for potent drug relief.

The literature provides evidence for the physical space in which health care is practiced - including factors such as light, music, architecture and colour - influencing the impact of the healing environment. Aesthetically pleasing and appropriately constructed buildings have been shown to help reduce anxiety, pain and infection rates in patients. The use of colour, texture and form to create pleasing environments and the creation of more homelike environments are recognised as important components of treatment and healing [20] and potential environments in which to promote health promoting behaviours. Despite the considerable research supporting both the benefits of affirmative design in the internal and external environments of health care facilities, there is sparse data relating to Australian Aboriginals. Further, based on the findings of this study, it is evident that the physical environment is only a part of the solution in improving health care for Aboriginal people [9,11]. Others have commented upon consumers identifying a need for changes in attitude and roles with a need to shift from one-off paternalistic attitudes of staff towards partnerships that really engage patients effectively in their care [24]. The attitudes expressed by our Aboriginal participants are not unique to them, but reflected broadly in other literature.

### A way forward

In order to address health disparities, a dedicated effort to engage Aboriginal people in sustainable and culturally appropriate cancer service planning is needed, an initiative successful in other areas of Aboriginal health care [7,26]. This raises the question of whether it requires that Aboriginal people have a better understanding of western views of cancer and modern cancer treatments. Given the Aboriginal view of health as extending beyond the individual and encompassing: "spiritual, cultural, emotional and social well-being (achieved through)... healthy, interdependent relationships between families, communities, land, sea and spirit" (quoted in, the views of participants are not surprising. Participants' responses also pose the question whether non-Aboriginal health care providers engage sufficiently with an Aboriginal approach to health care. Geffen argues that optimal cancer care balances the need for scientific knowledge and rational thought with the need for wisdom, kindness, compassion and love - "what people ultimately want most from their healing environment is meticulous medical care delivered with genuine love, caring and compassion. They want to be seen, respected, and accepted for who they are as individuals. These factors are far more important than the physical trappings of the centre...". Geffen's comments were not made in relation to Aboriginal people, but emphasize that improved provision of care for Aboriginal people will have flow-on benefits for all patients. It is perhaps unsurprising that so many of the issues raised by participants are already documented as design principles which impact on psychosocial and physical health outcomes [19].

The context of service planning to establish or expand a service, inevitably comes with a focus on issues related to architecture and building. Critical issues did emerge in our analysis that could be addressed by hospital planners: hospital rooms that accommodated large families; the opportunity for families to stay in safe, low cost accommodation close to the treatment centre and ready accessibility by public transport and parking; the location of an Aboriginal Welcome Reception Area at the entrance to the hospital with Aboriginal staff available to assist patients and family members effectively negotiate the hospital system; improved signage; considerations around privacy and sensitivity to gender issues. There was also a case for a building aesthetic that is more sympathetic to Aboriginal people in ambience, enabling views over or connection to Australian bushland. Many issues that relate to the buildings where cancer treatments occurred were raised in interviews, but they were overwhelmed by those that related to the social, family and human aspects of caring, including better linkages to primary care. It will remain essential to ensure good links and transition with primary health services and appropriate cancer support. The more recent announcement by the Australian Government of regional cancer units to provide care closer to home for rural citizens is very welcome and particularly likely to benefit Aboriginal people provided the design and service aspects incorporate the aspects of design and service delivery as described by them and captured here.

## Conclusions

The overwhelming dialogue around what respondents wanted from hospital services focused on feeling safe and cared for in an environment where the person felt the health team cared about them personally and understood their need to have family support. The primary tool for facilitating entry into and managing effective treatment within the current medical system is culturally sensitive person-to-person contact, support for Aboriginal family structures and a respect for the importance of place and community to Aboriginal patients. While building trust is more challenging than shaping bricks and mortar, the two are closely connected in a multi-tiered approach to improving experiences in hospital for Aboriginal people with cancer. A welcoming environment is evident from the moment a person arrives at the hospital and is reinforced by many features of design that impact upon the atmosphere and affect service provision. Designing the built environment to reflect these perspectives symbolises an acknowledgement of and respect for meeting the needs of Aboriginal patients that forms part of the healing process.

## Competing interests

The authors declare that they have no competing interests.

## Authors' contributions

SCT coordinated the whole project, participated in the design and assisted with the conduct of the study, analysis and writing. SS participated in the project's design, carried out the data collection and analysis for this project, and assisted with comments upon the draft. DB was involved in the data analysis phase and writing. AD helped interpret findings, and commented upon drafts of the manuscript. PMD helped interpret findings, and commented upon drafts of the manuscript. All authors read and approved the final manuscript.
